# Single-Base Genome Editing in *Corynebacterium glutamicum* with the Help of Negative Selection by Target-Mismatched CRISPR/Cpf1

**DOI:** 10.4014/jmb.2006.06036

**Published:** 2020-08-13

**Authors:** Hyun Ju Kim, Se Young Oh, Sang Jun Lee

**Affiliations:** Department of Systems Biotechnology, Chung-Ang University, Anseong 17546, Republic of Korea

**Keywords:** CRISPR/Cpf1, *Corynebacterium glutamicum*, single-base genome editing, target-mismatched crRNA, mismatch tolerance

## Abstract

CRISPR/Cpf1 has emerged as a new CRISPR-based genome editing tool because, in comparison with CRIPSR/Cas9, it has a different T-rich PAM sequence to expand the target DNA sequence. Single-base editing in the microbial genome can be facilitated by oligonucleotide-directed mutagenesis (ODM) followed by negative selection with the CRISPR/Cpf1 system. However, single point mutations aided by Cpf1 negative selection have been rarely reported in *Corynebacterium glutamicum*. This study aimed to introduce an amber stop codon in *crtEb* encoding lycopene hydratase, through ODM and Cpf1-mediated negative selection; deficiency of this enzyme causes pink coloration due to lycopene accumulation in *C. glutamicum*. Consequently, on using double-, triple-, and quadruple-basemutagenic oligonucleotides, 91.5–95.3% pink cells were obtained among the total live *C. glutamicum* cells. However, among the negatively selected live cells, 0.6% pink cells were obtained using single-base-mutagenic oligonucleotides, indicating that very few single-base mutations were introduced, possibly owing to mismatch tolerance. This led to the consideration of various targetmismatched crRNAs to prevent the death of single-base-edited cells. Consequently, we obtained 99.7% pink colonies after CRISPR/Cpf1-mediated negative selection using an appropriate singlemismatched crRNA. Furthermore, Sanger sequencing revealed that single-base mutations were successfully edited in the 99.7% of pink cells, while only two of nine among 0.6% of pink cells were correctly edited. The results indicate that the target-mismatched Cpf1 negative selection can assist in efficient and accurate single-base genome editing methods in *C. glutamicum*.

## Introduction

*Corynebacterium glutamicum*, a gram-positive, aerobic, soil-dwelling bacterium, is generally regarded as safe (GRAS), and is widely used for the industrial production of amino acids [1-3]. Since numerous chemicals, materials, and biofuels can be synthesized from biomass via simple metabolic pathways, *C. glutamicum* has received increasing attention as a robust and versatile cell factory [4, 5]. Owing to the low transformation efficiency of *C. glutamicum* when compared to *E. coli*, genetic or genome engineering of this bacterium is a limitation with respect to its industrial applications [6].

CRISPR technology initially emerged as a prokaryotic adaptive immune system [7-9] and has been recently developed as an efficient in vivo mutagenesis method in various microbial strains including *C. glutamicum* [10, 11]. Cas proteins along with crRNAs can together recognize and cleave target DNAs with a specific protospacer-adjacent motif (PAM) sequence. Upon introduction of mutagenic oligonucleotides to alter the target DNA sequence, the Cas/crRNA complex can cleave the unedited DNA targets, thus rendering genome-edited cells alive; this process is called negative selection [12-14]. Therefore, the ratio of edited cells to unedited cells aided by CRISPR/Cas-mediated negative selection is markedly higher than that of homology-directed repair (HDR) and classical oligo-directed mutagenesis [15-17].

CRISPR/Cas9 cleaves DNA target sequences with its 5′-NGG PAM sequence, yielding blunt ends [18]. *cas9* expression reportedly retards growth of *C. glutamicum* probably owing to the toxicity of gene products [19]. However, it has been known that CRISPR/Cpf1 from *Francisella novicida* displayed lower toxicity in *C. glutamicum* cells [17]. CRISPR/Cpf1 can recognize and cleave the target DNAs with different PAM sequence (5’-TTN), which is useful for engineering of A+T rich region [20-22].

Single oligonucleotide-directed mutagenesis with the coexpression of RecT recombinase has been developed as a rapid and efficient genome editing tool [23]. Two consecutive nucleotides have been successfully edited in the genome of *C. glutamicum* with very high efficiencies (86 to 100%) [17, 24]. However, single point mutations have been rarely reported in *C. glutamicum*. Single-base genome editing techniques that manipulate promoter strength and alter specific amino acid residues in cellular proteins are essential for future biotechnology. Recently, we reported efficient oligonucleotide-directed single base editing methods in *Escherichia coli*, using target-mismatched sgRNAs (single-molecular guide RNAs) during negative selection via CRISPR/Cas9 [25].

In this study, we tried to use target-mismatched CRISPR/Cpf1 system to change a single nucleotide in the genome of *C. glutamicum*. To monitor base editing through colony color change, we selected the *crtEb* gene that is involved in the biosynthesis of carotenoid pigments in *C. glutamicum*. Our results showed that the target-mismatched CRISPR/Cpf1 negative selection method helped efficient and accurate single-base editing in the *C. glutamicum* genome.

## Materials and Methods

### Bacterial Strains and Culture Conditions

The bacterial strains used herein are listed in Table S1. *E. coli* DH5α was cultured in Luria-Bertani (LB) broth at 37°C. *C. glutamicum* ATCC13869 was cultured in brain-heart infusion (BHI) broth at 30°C. Antibiotic concentrations, wherever necessary, were 25 μg/ml kanamycin, 50 μg/ml spectinomycin, and 25 μg/ml chloramphenicol for *E. coli* and 25 μg/ml kanamycin, 75 μg/ml spectinomycin, and 25 μg/ml chloramphenicol for *C. glutamicum*.

### Plasmid Constructions

All plasmid constructs used herein are listed in Table S1. Furthermore, the primers and oligonucleotides used herein are listed in Table S2. Genomic DNA was extracted using the Wizard Genomic DNA purification kit (Promega A2611, USA). Plasmids and PCR products were extracted using the NucleoSpin Plasmid EasyPure kit (Macherey-Nagel, Germany, Cat No. 740727) and the NucleoSpin Gel and PCR Clean-up kit (Macherey-Nagel, Cat No. 740609), respectively. KOD FX polymerases were used for PCR (Toyobo, Japan, Cat No. KFX-101). DNA fragments were amplified and assembled to generate various plasmids, using the Gibson assembly mix (NEB, Hitchin, UK, Cat No. E2611).

To generate the *cpf1* integration pHK487 plasmid (10.3 kb), we assembled three DNA fragments amplified as follows: first, pK18mobSacB [26] was used as a template to amplify a 5.7-kb DNA fragment, using P1/P2 primers. Second, a homologous region (0.5 kb) of *C. glutamicum* ATCC13869 genome (*cg1121*) was amplified using *C. glutamicum* ATCC13869 genomic DNA as a template and P3/P4 primers. The third DNA fragment (4.0 kb) of *cpf1* under the control of the *P_lacM_* promoter was obtained through PCR amplification of pJYS1Ptac plasmid with P5/P6 primers.

To generate the RecT expression vector (pHK489), we first constructed a Cpf1-deleted vector (pHK432). pJYS2_crtYf plasmid was used as a template to amplify a ~8.1-kb fragment using 5′-phosphorylated primers P7/P8. This fragment was treated with DpnI and self-ligated to construct pHK432. Thereafter, a ~7.4-kb fragment was amplified using pHK432 as a template and P9/P10 primers. A 0.7-kb fragment of the chloramphenicol resistance gene was amplified using pSL360 [27] as a template and P11/P12 primers. These two fragments (~7.4 and 0.7 kb) were purified and recovered for isothermal assembly.

To introduce temperature-sensitive origin of replication in the crRNA expression vector, pJYS1Ptac plasmid was used as a template to amplify a ~1.5-kb fragment of pBL *ts-ori* using P13/P14 primers. A ~2.9-kb crRNA backbone was amplified using pJYS2_crtYf plasmid as a template and P15/P16 primers. These two fragments were purified and recovered for isothermal assembly to generate pHK473.

To construct perfect-matched and mismatched crRNA expression plasmids targeting *crtEb*, pHK473 was used as a template to amplify a ~1.9-kb fragment and a ~2.5-kb fragment. These two fragments were digested with DpnI and purified for isothermal assembly to generate pHK493. Other crRNA expression vectors (pHK494–pHK499) were generated using the same method as that used for pHK493 and confirmed through Sanger sequencing, using P35.

To generate crRNA-deleted plasmid (pHK475) for analyzing the transformation efficiency of HK1220/pHK489 competent cells, pHK473 was used as a template to amplify a ~8.0-kb fragment using 5′-phosphorylated P17/P18 primers. This fragment was treated with DpnI and self-ligated to construct pHK475.

### Electrocompetent Cells

Electrocompetent *C. glutamicum* cells were generated as previously described with minor modifications.[17] *C. glutamicum* ATCC13689 and its derivatives were cultured on BHI agar. A single colony from each strain was inoculated into 15 ml of BHI supplemented with 50 mM sorbitol and 10 g/l glucose (BHISG), and cultured overnight at 30°C with agitation at 200 rpm. If needed, chloramphenicol (25 μg/ml) was added in the culture. From this pre-culture, 10 ml was inoculated into 200 ml of BHISG supplemented with 1 ml/l Tween 80 and 4 g/l glycine. Cells harboring pHK489 were treated with 0.5 mM isopropyl β-D-1-thiogalactopyranoside (IPTG) for inducing RecT recombinase. Cells were harvested and rendered electrocompetent when the OD_600_ approached ~1.0. Cells were chilled on ice for 20 min and harvested through centrifugation at 3,500 rpm and 4°C for 20 min and washed thrice with 50 ml of ice-chilled 10% glycerol. Competent cells were resuspended 2 ml of 10 % glycerol, and 100-μl aliquots were stored at −80°C.

### Genome Editing

Plasmids (~2 μg) and oligonucleotides (1 μg) were added to the electrocompetent cells thawed on ice and then transferred into pre-cooled electroporation cuvettes and covered with 100 μl of 10% glycerol. Electroporation was performed at 25 μF, 200 Ω, and 2.5 kV, using 4°C precooled electroporation cuvette (width, 2 mm). Cells were immediately transferred to 800 μl of BHISG medium and heat-shocked for 6 min at 46°C. The cells were then allowed to recover for 3 h at 30°C with agitation at 180 rpm. Thereafter, recovered cells were spread on BHI containing chloramphenicol or spectinomycin and incubated for 72 h at 30°C. Pink colonies on agar plates were enumerated to determine the base editing efficiency, and nine colonies per plate were selected for Sanger sequencing.

## Results

### Construction of a CRISPR/Cpf1- and RecT-Mediated Scarless Genome Editing System 

**Fig. 1:**
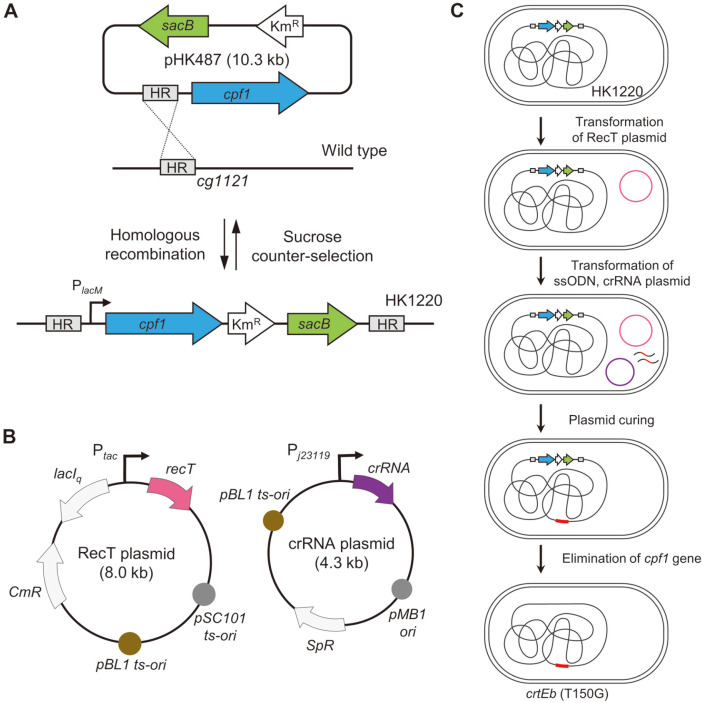
Schematic representation of the CRISPR/Cpf1 system in *Corynebacterium glutamicum*. (**A**) Chromosomal integration of *cpf1* in the *cg1121* locus via homologous recombination. (**B**) RecT expression plasmid and crRNA expression plasmid. (**C**) Scarless genome editing flow. RecT plasmid was electroporated into HK1220 cells. Mutagenic oligonucleotides and crRNA plasmid were electroporated into IPTG-induced HK1220/pHK489 cells. After genome editing, plasmids were cured through culturing at high temperatures and *cpf1* was eliminated via the sacB (encoding levansucrase) counter-selection system.

To stably express *cpf1* and generate a scarless genome editing system, we electroporated non-replicating plasmid pHK487 harboring *cpf1*, and confirmed the integration of pHK487 in the chromosome of *C. glutamicum* ATCC13869 strain, which was named the HK1220 strain (Fig. 1A). Subsequently, *recT*-expressing plasmid pHK489 was transformed into HK1220 cells, which were cultured, induced with IPTG, and harvested for electroporation of crRNA plasmids and mutagenic oligonucleotides (Fig. 1B). After obtaining genome-edited cells negatively selected using the CRISPR/Cpf1 system, plasmids were cured at 42°C, and *cpf1* was deleted by the counter-selectable sacB system, retaining only edited sequences in the genome of *C. glutamicum* (Fig. 1C).

### Oligonucleotide-Directed Genome Editing of *crtEb* in *C. glutamicum*

**Fig. 2:**
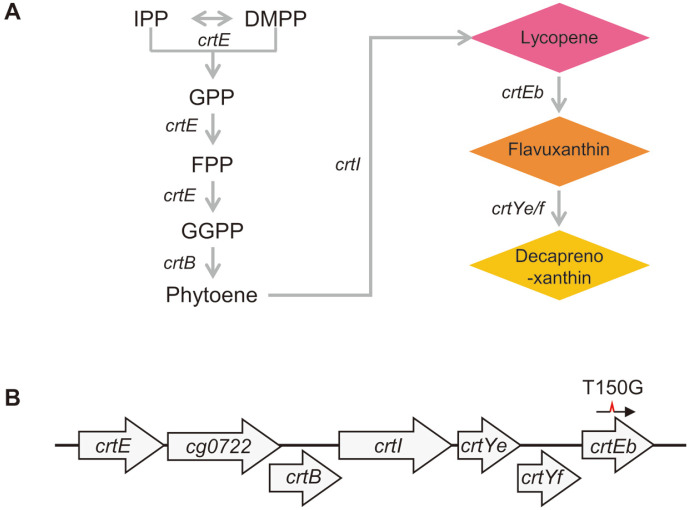
Carotenoid biosynthesis in *C. glutamicum*. (**A**) Decaprenoxanthin biosynthetic pathway and genes. IPP, isopentenyl pyrophosphate; DMPP, dimethylallyl pyrophosphate; GPP, geranyl pyrophosphate; FPP, farnesyl pyrophosphate; GGPP, geranylgeranyl pyrophosphate. (**B**) Structure of the crt operon in *C. glutamicum* ATCC13869.

*crtEb* in the carotenoid biosynthesis operon was selected as a target gene for base editing in the genome of *C. glutamicum*, the disruption of which resulted in the conversion of yellow to pink cells owing to lycopene accumulation in the impaired carotenoid synthesis pathway (Fig. 2) [28]. We introduced a stop codon (Y50Z) in the middle of *crtEb*, through oligonucleotide mutagenesis and subsequent negative selection via the CRISPR/Cpf1 system, and we then estimated the base editing efficiency by enumerating the pink colonies among all surviving cells after transformation of crRNA plasmids and mutagenic oligonucleotides.

**Fig. 3:**
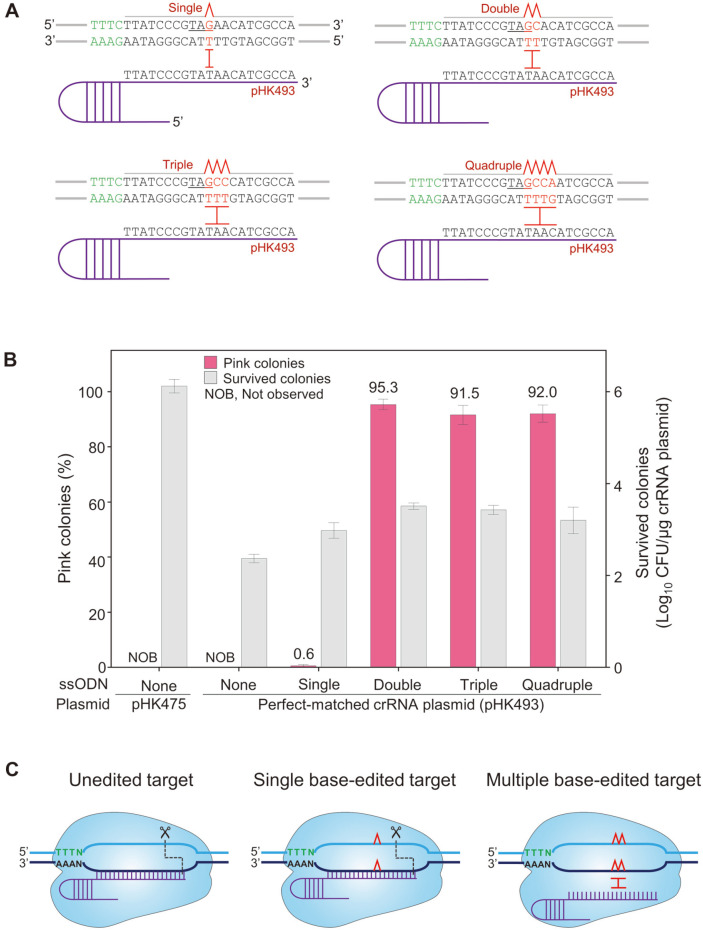
Negative selection of oligonucleotide-directed mutations in *crtEb* by CRISPR/Cpf1. (**A**) Negative selection of single-, double-, triple-, and quadruple-base edited targets in *crtEb* by target-matched CRISPR/Cpf1. Amber stop codon (TAG) generated through oligonucleotide-directed mutagenesis is underlined. (**B**) The proportion of pink colonies representing the apparent editing efficiency and the number of surviving colonies after electroporation of the target-matched crRNA plasmid (pHK493) and various mutagenic oligonucleotides. Each bar represents the average of three independent experiments. ssODN, single-stranded oligodeoxynucleotide. (**C**) Schematic representation of the cleavage of single-base-edited targets by CRISPR/Cpf1 owing to mismatch tolerance.

The crRNA plasmid pHK493 was electroporated with four different oligonucleotides inducing single (^150^T to G), double (^150^TA to GC), triple (^150^TAA to GCC), and quadruple (^150^TAAC to GCCA) base mutations in the genome, where a T150G transversion can cause a nonsense mutation (Y50Z) in *crtEb* (Fig. 3A). Consequently, without mutagenic oligonucleotides, no pink cells were obtained. Double-, triple-, and quadruple-base-edited pink cells were obtained with very high editing efficiencies of 95.3, 91.5, and 92.0%, respectively (Fig. 3B). However, single-base-edited pink cells were rarely observed with a low editing efficiency of 0.6%, where the shape and size of transformant colonies were not homogeneous (Fig. S1), indicating that CRISPR/Cpf1-mediated negative selection was not efficient with single-base mutagenic oligonucleotides, presumably because single base-edited target DNA sequences can be also cleaved as a target by the Cpf1/crRNA complex, which is known as mismatch tolerance (Fig. 3C) [29]. When more than two-mismatched base pairs were present between edited DNA target and crRNA, the target seems not to be recognized by Cpf1/crRNA complex.

In the case of single-base oligonucleotides, 3.0 × 10^2^ (CFU/μg DNA of pHK493) of transformant cells survived among the electrocompetent cells, with a transformation efficiency of ~10^6^ CFU/μg DNA of pHK475 (Fig. 3B). Even without mutagenic nucleotides, 2.4 × 10^2^ (CFU/μg DNA of pHK493) of transformant cells survived owing to failed negative selection with the CRISPR/Cpf1 system.

### Single-Base Genome Editing by Target-Mismatched crRNAs

**Fig. 4:**
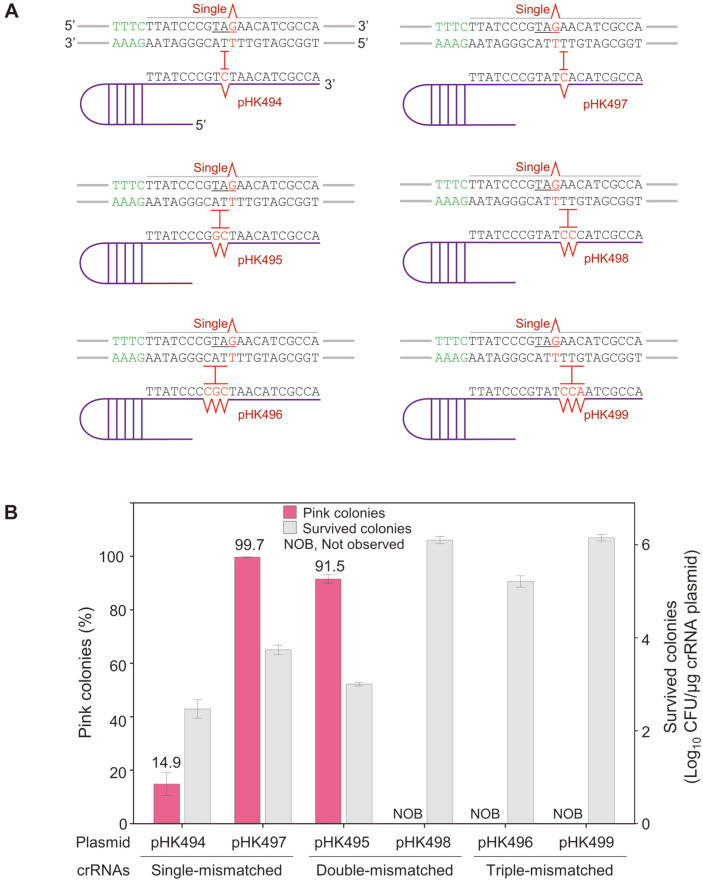
Single-base genome editing by target-mismatched CRISPR/Cpf1. (**A**) Design of target mismatched-crRNAs to prevent the cleavage of single-base-edited DNA targets. The amber stop codon (TAG) generated through oligonucleotide-directed mutagenesis is underlined. (**B**) The proportion of pink colonies representing the apparent editing efficiency and the number of surviving colonies after electroporation of various target-mismatched crRNA plasmids and single-base-mutagenic oligonucleotides. Each bar represents the average of three independent experiments.

Since double-, triple-, and quadruple-base mutations were successfully obtained through negative selection, target-mismatched crRNAs were designed to cleave unedited DNA without cleaving a single-base-edited DNA sequence. One- to three-base-mismatched crRNA plasmids along with single-base mutagenic oligonucleotides were electroporated into IPTG-induced HK1220/pHK489 cells for negative selection of single-base-edited DNA sequences (Fig. 4A). Consequently, in cases of single-mismatched crRNAs (pHK494 and pHK497), pink colonies were obtained with efficiencies of 14.9 and 99.7%, respectively. When double-mismatched crRNA (pHK495) were used, we obtained single-base edited pink colonies with an editing efficiency of 91.5%. At higher editing efficiencies, the shape and size of transformant colonies were more homogeneous (Fig. S3). When another double-mismatched (pHK498) and two triple-mismatched crRNAs (pHK496 and pHK499) were used, no pink colonies were observed at higher transformation efficiencies (~10^6^ CFU/μg DNA), indicating that those crRNAs cannot recognize even unedited DNA target sequences.

**Fig. 5:**
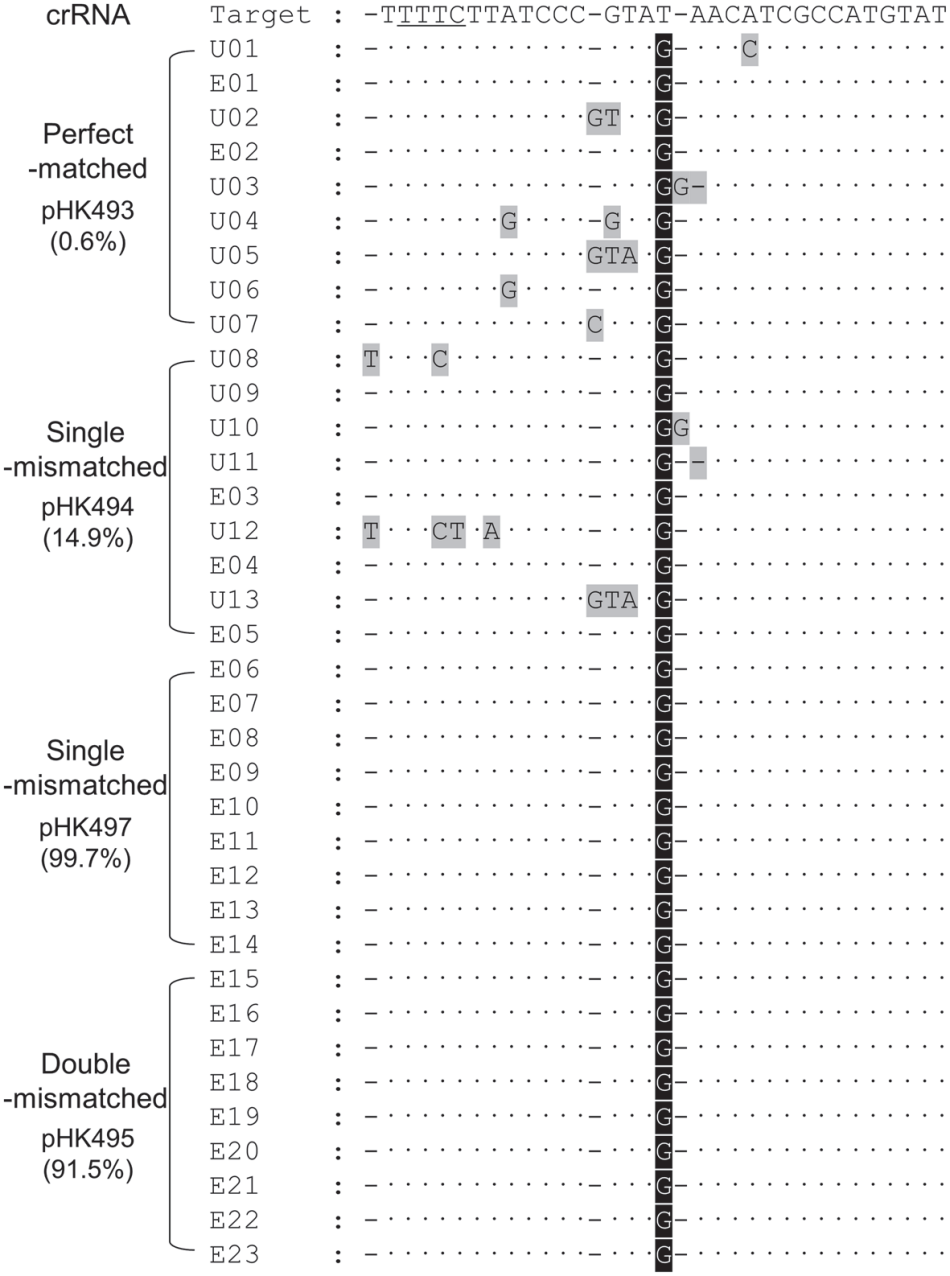
Sequence alignment of single-base-edited target regions in *crtEb*. The PAM sequence of Cpf1 was underlined. Dots and bars indicate perfectly aligned nucleotides and gaps, respectively, in comparison with the target DNA sequence. The gray-shaded nucleotides indicate undesirable mutations. The black-shaded G indicate single-base-edited nucleotides (T150G) after genome editing. E01–E23 show precise single-base changes, and U01–U13 show undesirable substitutions and indels proximal to the edited target region. Parenthesis indicate the proportion of pink colonies among the surviving colonies.

Furthermore, pink colonies were randomly selected from negatively selected pink colonies (pHK493, pHK494, pHK497, and pHK495), and Sanger sequencing was carried out for edited regions in *crtEb* (Fig. 5). Consequently, in case of perfectly matched crRNA (pHK493), two of nine regions were accurately altered. In the case of single-mismatched pHK494, three of nine regions were perfectly edited. In the case of pHK497 (single-mismatched) and pHK495 (double-mismatched), all T-to-G single-base edits were successful, as intended. These results indicated that the use of target-mismatched crRNA is not only an efficient but also an accurate negative selection method for single-base genome editing using the CRISPR/Cpf1 system.

## Discussion

Since bacterial cells synthesize valuable metabolites as encoded by their genomes, precise editing of microbial genomes is indispensable for the design of microbial cell factories. CRISPR/Cas9 (or Cpf1) technologies have been recently developed to edit genome sequences in numerous cellular platforms including *C. glutamicum* [30]. The PAM sequence (5′-NYTV) of CRISPR/Cpf1 [31] does not restrict editing of the genome of industrial *C. glutamicum* strains with G+C contents of 53.8% [32]. Moreover, owing to the potential toxicity of CRISPR/Cas9, the CRISPR/Cpf1 system of *C. glutamicum* has received increasing attention [11, 17, 33, 34].

Herein, we integrated *cpf1* in the *C. glutamicum* genome because frequent transformations were laborious for introducing a genome editing tool into *C. glutamicum* with a low transformation efficiency. The intergenic region between *cg1121*-cg1122 has been often used as an integration site of foreign genes in *C. glutamicum* genome [35, 36], which might not affect cellular growth and/or physiology of *C. glutamicum*. First, *cpf1* was introduced into the *cg1121*-cg1122 intergenic region of *C. glutamicum* ATCC13869 genome via a single-crossover to generate strain HK1220 (Fig. 1A). *recT* products facilitate the oligonucleotide-directed homologous recombination in *C. glutamicum* (Fig. 1B) [37]. After genome engineering, plasmids can be eliminated through high temperature treatment, and *cpf1* can be excised from the HK1220 genome, using a counter-selectable sacB marker (Fig. 1C).

Negative selection using CRISPR/Cpf1 facilitates the survival of genome-edited cells; however, unedited cells are eliminated through double-stranded breaks at target DNA sequences. Therefore, CRISPR/Cpf1 can increase the editing efficiency of surviving cells [38]. After transformation of IPTG-induced HK1220 cells harboring the RecT plasmid (pHK489) with single-stranded mutagenic oligonucleotides and crRNA plasmids, the surviving cells putatively harboring the desired mutations were obtained through negative selection (Fig. 3A). The use of double, triple, and quadruple mutagenic oligonucleotides successfully introduced the TAG stop codon in the middle of *crtEb* with an editing efficiency (*i.e.*, the proportion of pink colonies) of 91.5–95.3%. However, we rarely obtained pink colonies (0.6%) when using single-base-mutagenic oligonucleotides (Fig. 3B). Moreover, Sanger sequencing revealed successful single-base edits in 2 of 9 selected pink colonies generated through target-matched crRNA (pHK493) (Fig. 5), probably owing to mismatch tolerance, whereby the Cpf1/crRNA complex can recognize and cleave both single-base-edited and unedited targets (Fig. 3C), which have been assessed to resolve off-target effects [29, 39, 40].

Even upon transformation of only crRNA plasmids into cells without oligonucleotides, we still observed numerous surviving cells (~10^2^ CFU/μg DNA of pHK493), probably owing to null *cpf1* mutations or the repair of double-strand breaks in target DNA sequences. Furthermore, heterogeneity in colony shape and size was observed primarily in cases of failed negative selection (Figs. S1 and S2). Accurately edited colonies were larger than unedited colonies in our Cpf1-mediated study, while larger colonies were false-positive during Cas9-mediated genome editing of *C. glutamicum* [33]. Therefore, colony size does not reflect successful genome editing on using CRISPR-mediated negative selection.

To differentiate single-base-edited targets from unedited targets, mismatched crRNAs were designed and used for precise CRISPR/Cpf1-mediated negative selection (Fig. 4A). With single-base-mutagenic oligonucleotides, different target-mismatched crRNA plasmids were transformed for single-base editing of T150G (*i.e.*, introduction of TAG stop codon) in *crtEb*. In cases of single-mismatched crRNAs (from pHK494 and pHK497), and one of double-mismatched crRNAs (from pHK495), 14.9%, 99.7%, and 91.5% of surviving colonies were pink owing to intracellular lycopene accumulation (Fig. 4B). Subsequently, pink colonies were randomly selected from each agar plate and their genomes were subjected to Sanger sequencing. Three of nine colonies were correctly edited among 14.9% pink colonies. The DNA sequences were accurately edited in all colonies among 99.7% and 91.5% of surviving colonies (Fig. 5), indicating that even if mutants harboring the desired phenotypes were obtained among the colonies obtained through negative selection, using the CRISPR/Cpf1 system, the efficiency of harboring a genotype that is precisely altered to the base sequence can be much lower.

The transformation efficiencies reflected between 10^2^ and 10^4^ CFU/μg crRNA plasmid among genome-edited cells. However, in one case of double- and two cases of triple-mismatched crRNAs (from pHK498, pHK496, and pHK499), no pink colonies were observed. Moreover, the number of surviving colonies increased to 10^5^–10^6^ (CFU/μg crRNA plasmid). The increased number of surviving colonies indicates that Cpf1/target-mismatched crRNAs could not accurately recognize the targets, and consequently, improper negative selection facilitated the survival of all transformants on agar plates. As applicable design rules for target-mismatched sgRNAs in CRISPR/Cas9 system have been provided [25], further studies should address how to design mismatched crRNAs in CRISPR/Cpf1 for single base editing in microbial genomes.

In summary, single-base genome editing is indispensable for repairing errors in nucleotide sequences in microbial cell factories. Moreover, useful genotypes representing evolved phenotypes can be introduced directly into new backgrounds through precise base editing methods. For example, promoter strength and/or transcriptional regulatory sequences can be altered, and codons of interest in the structural gene can be also edited. The target-mismatched crRNA method is an efficient negative selection tool for elaborate single base editing in *C. glutamicum*, which could be extended to other platform microbial cells.

## Supplemental Materials



Supplementary data for this paper are available on-line only at http://jmb.or.kr.
