# Supplementary material for: Single-Base Genome Editing in Corynebacterium glutamicum with the Help of Negative Selection by Target-Mismatched CRISPR/Cpf1
Source: J Microbiol Biotechnol. 2020 Aug 13;30(10):1583–91. doi: 10.4014/jmb.2006.06036 (PMC9728170; doi:10.4014/jmb.2006.06036)
Supplement: Supplementary file 1 [file JMB-30-10-1583-supple.pdf]

J. Microbiol. Biotechnol.
https://doi.org/10.4014/jmb.2006.06036

Table S1. Bacterial strains and plasmids used in this study.

Name	Characteristics	Reference or source
**Bacterial strains**		
Escherichia coli		
DH5α	*F-eNDA1 glnV44 thi-1 recA1 relA1 gyrA96 deoR nupG Φ80dlacZΔM15 Δ*(*lacZYA-argF*)*U169*, *hsdR17* (*rK-mK*),*λ–*	Laboratory stock
Corynebacterium glutamicum		
ATCC 13869	Wild type	American Type Culture Collection (ATCC)
HK1220	Single cross-over integration of the *cpf1* gene in ATCC 13869	This study
**Plasmids**		
pK18mobsacB	*sacB*, *lacZa*, Km^r, mcs mobilizable vector, allows for selection of double crossover *C. glutamicum*	[1]
pJYS1Ptac	pBL1^{ts} $oriV_{C.glutamicum}$ Km^r pSC101 $oriV_{E.\ coli}$ *lacIq*, PlacM-*Fn*Cpf1, Ptac-RecT	Addgene #85545
pJYS2_crtYf	*rep* $oriV_{C.glutamicum}$ Sp^r pMB1 $oriV_{E.\ Coli}$ Pj23119-crRNA targeting *crtYf*	Addgene #85544
pSL360	pSK1Cat carrying P180	[2]
pHK432	Deletion of *cpf1* gene from pJYS1Ptac	This study
pHK473	pBL1^{ts} $oriV_{C.\ glutamicum}$ Sp^r pMB1 $oriV_{E.\ coli}$ Pj23119-crRNA targeting *crtYf*	This study
pHK475	Deletion of crRNA region from pHK473	This study
pHK487	pK18mobsacB containing HR1 and the *cpf1* gene for integration between cg1121 and cg1122	This study
pHK489	pBL1^{ts} $oriV_{C.glutamicum}$ Cm^r pSC101 $oriV_{E.\ coli}$ *lacIq*, Ptac-RecT	This study
pHK493	pBL1^{ts} $oriV_{C.\ glutamicum}$ Sp^r pMB1 $oriV_{E.\ coli}$ Pj23119-crRNA targeting *crtEb* (^{140}TTATCCCGTATAACATCGCCA^{160})	This study
pHK494	pBL1^{ts} $oriV_{C.\ glutamicum}$ Sp^r pMB1 $oriV_{E.\ coli}$ Pj23119-crRNA targeting *crtEb* (^{140}TTATCCCGTCTAACATCGCCA^{160})	This study
pHK495	pBL1^{ts} $oriV_{C.\ glutamicum}$ Sp^r pMB1 $oriV_{E.\ coli}$ Pj23119-crRNA targeting *crtEb* (^{140}TTATCCCGGCTAACATCGCCA^{160})	This study
pHK496	pBL1^{ts} $oriV_{C.\ glutamicum}$ Sp^r pMB1 $oriV_{E.\ coli}$ Pj23119-crRNA targeting *crtEb* (^{140}TTATCCCCGCTAACATCGCCA^{160})	This study
pHK497	pBL1^{ts} $oriV_{C.\ glutamicum}$ Sp^r pMB1 $oriV_{E.\ coli}$ Pj23119-crRNA targeting *crtEb* (^{140}TTATCCCGTATCACATCGCCA^{160})	This study
pHK498	pBL1^{ts} $oriV_{C.\ glutamicum}$ Sp^r pMB1 $oriV_{E.\ coli}$ Pj23119-crRNA targeting *crtEb* (^{140}TTATCCCGTATCCCATCGCCA^{160})	This study
pHK499	pBL1^{ts} $oriV_{C.\ glutamicum}$ Sp^r pMB1 $oriV_{E.\ coli}$ Pj23119-crRNA targeting *crtEb* (^{140}TTATCCCGTATCCAATCGCCA^{160})	This study

Table S2. Primers used in this study.

Name	Sequence (5'→3')	Features
**Mutagenic oligonucleotides (T150G at the *crtEb* gene for stop codon)**		
59mer-S	GTAGGCATAGTATTTTTTCTTATCCCGTAGAACATCGCCATGTATGGCATCAACGATGT	Single-mutagenic (^{150}T to G)
59mer-D	GTAGGCATAGTATTTTTTCTTATCCCGTAGCACATCGCCATGTATGGCATCAACGATGT	Double-mutagenic (^{150}TA to GC)
59mer-T	GTAGGCATAGTATTTTTTCTTATCCCGTAGCCCATCGCCATGTATGGCATCAACGATGT	Triple-mutagenic (^{150}TAA to GCC)
59mer-4	GTAGGCATAGTATTTTTTCTTATCCCGTAGCCAATCGCCATGTATGGCATCAACGATGT	Quadruple-mutagenic (^{150}TAAC to GCCA)
**Construction of the Cpf1 integrated vector**		
P1	CAATAACTAAGTCCCTTTGAGTGAGCTGATACCGCTCGCCG	Construction of pHK487
P2	CAAGAACCAGGACCGGTAATACGGTTATCCACAGAATCAGG	Construction of pHK487
P3	AACCGTATTACCGGTCCTGGTTCTTGTCCTGGGCAACGTTG	Construction of pHK487
P4	GATTCCGCGAACCCCAGAGTCCCGCAGGAGCCTCAAAAATCGAGCTCGCTTTGGTC	Construction of pHK487
P5	CAAAGCGAGCTCGATTTTTGAGGCTCCTGCGGGACTCTGGGGTTCGCGGAATCATG	Construction of pHK487
P6	GCTCACTCAAAGGGACTTAGTTATTGCGGTTCTGGACAAAT	Construction of pHK487
**Construction of the RecT expression vector**		
P7	[5'-Phosphorylated] CACACATGGTACCACACGATGATTAATTG	Construction of pHK432
P8	[5'-Phosphorylated] GAAAGGCCCAGTCTTTCGACTGAGCCTTTC	Construction of pHK432
P9	TTTTTTTCTCCATGCGAAACGATCCTCATCCTGTCTCTTGA	Construction of pHK489
P10	GCAGGGCGGGGCGGCGGGACTCTGGGGTTCGCGGAATCATG	Construction of pHK489
P11	AGGATCGTTTCGCATGGAGAAAAAAATCACTGGATATACCA	Construction of pHK489
P12	CCCAGAGTCCCGCCGCCCCGCCCTGCCACTCATCGCAGTAC	Construction of pHK489
**Construction of the crRNA expression vector**		
P13	GAAAGCACTCAAGACGTGCGAGCTACCAACTCATATGCACGGG	Construction of pHK473
P14	CGGTGTTTCGTTTAGTGGGTGCGAAGAATAGTCTGCTCATTAC	Construction of pHK473
P15	ATTCTTCGCACCCACTAAACGAAACACCGTCAGCAGAAAACGG	Construction of pHK473
P16	GTTGGTAGCTCGCACGTCTTGAGTGCTTTCTCCCAGCTGATGAC	Construction of pHK473
P17	[5'-Phosphorylated] TTCGAGTCGAGGAAGAGCCAGAGCAGAAGGC	Construction of pHK475
P18	[5'-Phosphorylated] TTCGTTTGTCGGTGAACGCTCTCCTGAGTAG	Construction of pHK475
P19	GATATTCCATTTTCTGATGTGAGAAGAGCCATTATGGATTC	Construction of crRNA plasmids

P20	CTTCTCACATCAGAAAATGGAATATCAGGTAGTAATTCCTC	Construction of crRNA plasmids
P21	TTATCCCGTATAACATCGCCAATTTAAATAAAACGAAAGGCTCAG	Construction of pHK493
P22	TGGCGATGTTATACGGGATAAATCTACAACAGTAGAAATTCGGAT	Construction of pHK493
P23	TTATCCCGTCTAACATCGCCAATTTAAATAAAACGAAAGGCTCAG	Construction of pHK494
P24	TGGCGATGTTAGACGGGATAAATCTACAACAGTAGAAATTCGGAT	Construction of pHK494
P25	TTATCCCGGCTAACATCGCCAATTTAAATAAAACGAAAGGCTCAG	Construction of pHK495
P26	TGGCGATGTTAGCCGGGATAAATCTACAACAGTAGAAATTCGGAT	Construction of pHK495
P27	TTATCCCCGCTAACATCGCCAATTTAAATAAAACGAAAGGCTCAG	Construction of pHK496
P28	TGGCGATGTTAGCGGGGATAAATCTACAACAGTAGAAATTCGGAT	Construction of pHK496
P29	TTATCCCGTATCACATCGCCAATTTAAATAAAACGAAAGGCTCAG	Construction of pHK497
P30	TGGCGATGTGATACGGGATAAATCTACAACAGTAGAAATTCGGAT	Construction of pHK497
P31	TTATCCCGTATCCCATCGCCAATTTAAATAAAACGAAAGGCTCAG	Construction of pHK498
P32	TGGCGATGGGATACGGGATAAATCTACAACAGTAGAAATTCGGAT	Construction of pHK498
P33	TTATCCCGTATCCAATCGCCAATTTAAATAAAACGAAAGGCTCAG	Construction of pHK499
P34	TGGCGATTGGATACGGGATAAATCTACAACAGTAGAAATTCGGAT	Construction of pHK499
P35	GAGCTTGCAGGCGAACTAGGTGTCAGTG	Sequencing of crRNA plasmids
**Cpf1 integration in the *C. glutamicum* genome**		
P36	CAGAACCCATACGGTGGTTACGACAAC	Upstream of HR region
P37	GTTGGCTCCGTTGTTCACGTCTACTACG	Downstream of HR region
P38	GAGCTCTTCGGCCAGGTCCTTCTTGATC	Reverse primer of the *cpf1* gene
P39	CGAGACCTTCAAGAAGATGGGCAAGCAG	Forward primer of the *cpf1* gene
**Sequencing of *crtEb***		
P40	CTTCTGATTCCTGCCCTATGGTTGCCTG	Sequencing of the edited target
P41	GTTGTGGAGCTTGAACGCATCGAGGTCG	Sequencing of the edited target

Figure S1. Transformation agar plates after electroporation of single-, double-, triple-, and quadruple-base mutagenic oligonucleotides and perfect-matched crRNA (pHK493) plasmid into IPTG-induced HK1220/pHK489 cells.

Single base mutagenic oligo, perfect-matched crRNA (pHK493)	Double base mutagenic oligo, perfect-matched crRNA (pHK493)
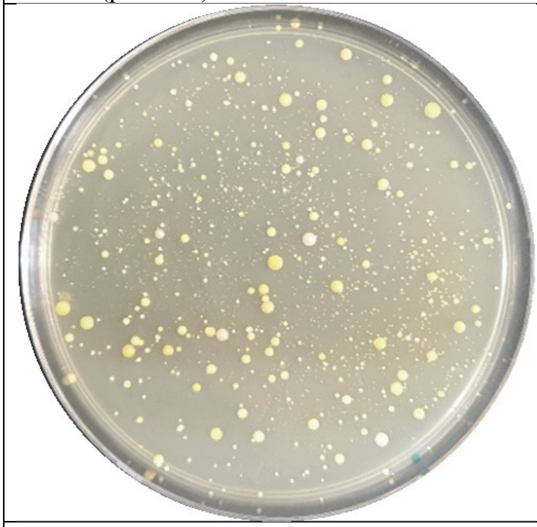	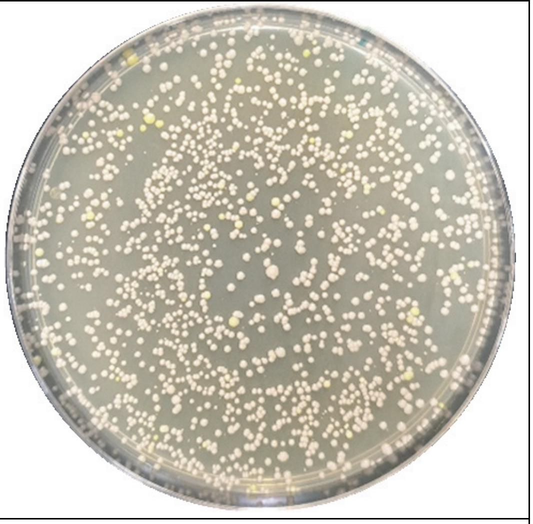
Triple base mutagenic oligo, perfect-matched crRNA (pHK493)	Quadruple base mutagenic oligo, perfect-matched crRNA (pHK493)
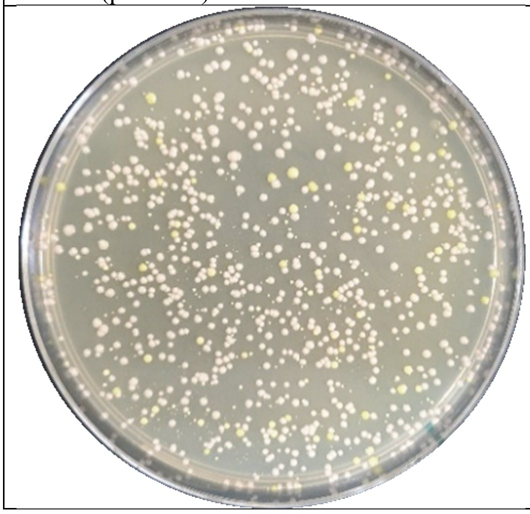	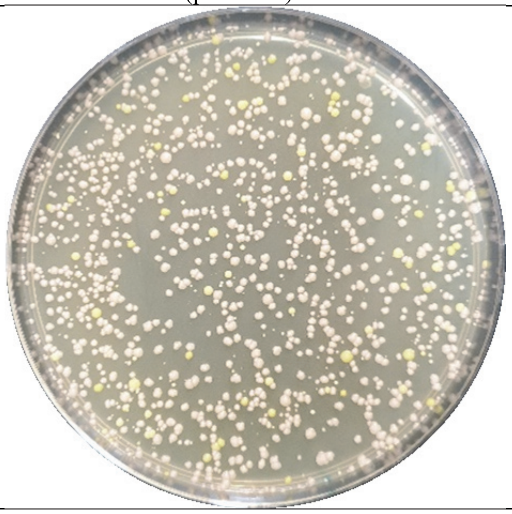

Figure S2. Transformation agar plates after electroporation of single-base-mutagenic oligonucleotides and mismatched crRNA plasmids into IPTG-induced HK1220/pHK489 cells.

Single base mutagenic oligo, single-mismatched crRNA (pHK494)	Single base mutagenic oligo, single-mismatched crRNA (pHK497)
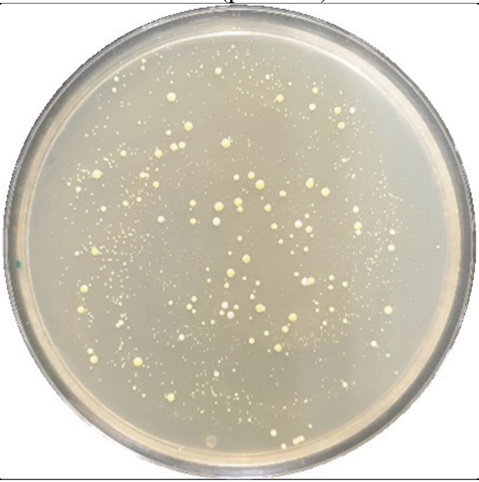	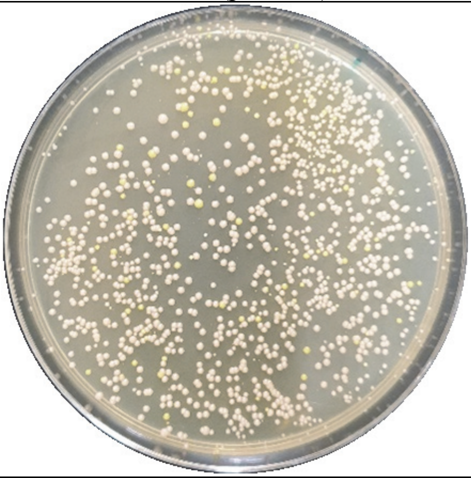
Single base mutagenic oligo, double-mismatched crRNA (pHK495)	
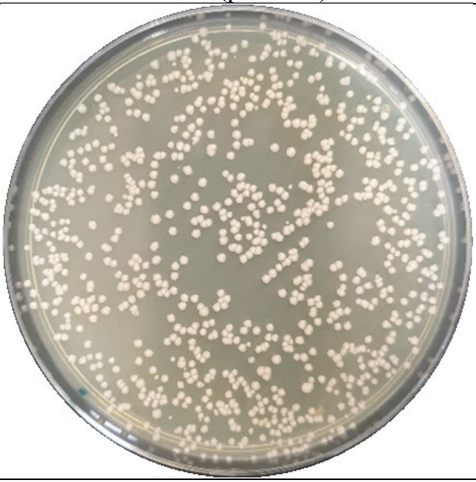	

Figure S3. Chromatogram of the edited sequences of *crtEb*.

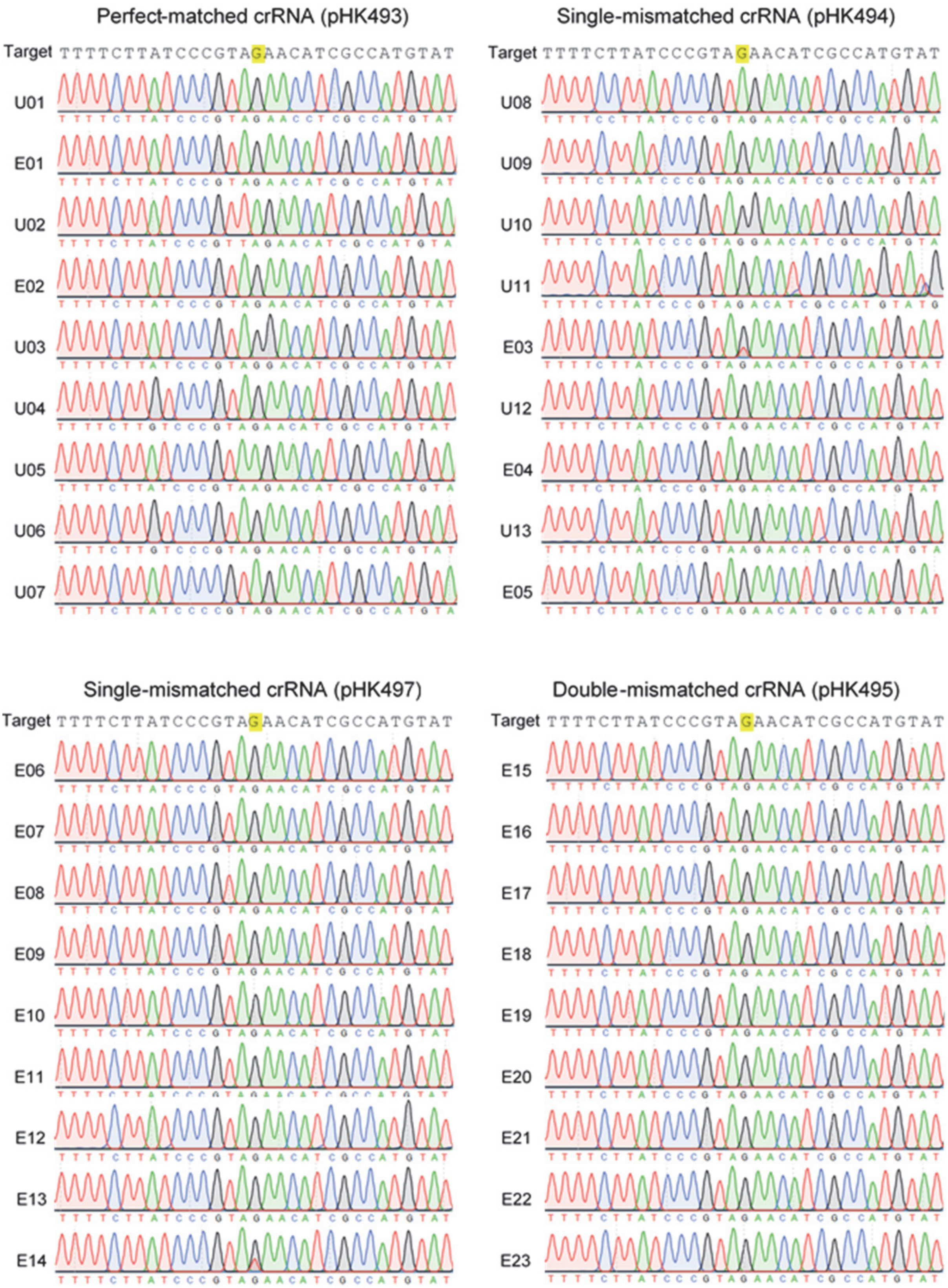

References

1. Schafer A, Tauch A, Jager W, Kalinowski J, Thierbach G, Puhler A: **Small mobilizable multi-purpose cloning vectors derived from the *Escherichia coli* plasmids pK18 and pK19: selection of defined deletions in the chromosome of *Corynebacterium glutamicum*.** *Gene* 1994, **145:**69-73.
2. Park S-D, Lee S-N, Park I-H, Choi J-S, Jeong W-K, Kim Y, Lee H-S: **Isolation and characterization of transcriptional elements from *Corynebacterium glutamicum*.** *Journal of Microbial Biotehcnology* 2004, **14:**789-795.
